# Hyperpolarised xenon magnetic resonance spectroscopy for the longitudinal assessment of changes in gas diffusion in IPF

**DOI:** 10.1136/thoraxjnl-2018-211851

**Published:** 2018-11-02

**Authors:** Nicholas D Weatherley, Neil J Stewart, Ho-Fung Chan, Matthew Austin, Laurie J Smith, Guilhem Collier, Madhwesha Rao, Helen Marshall, Graham Norquay, Stephen A Renshaw, Stephen Mark Bianchi, Jim M Wild

**Affiliations:** 1 POLARIS, Academic Unit of Radiology, University of Sheffield, Sheffield, UK; 2 Academic Directorate of Respiratory Medicine, Sheffield Teaching Hospitals Foundation Trust, Sheffield, UK; 3 Department of Infection, Immunity and Cardiovascular Disease, University of Sheffield, Sheffield, UK

**Keywords:** idiopathic pulmonary fibrosis, interstitial fibrosis, imaging/ct mri etc, lung physiology

## Abstract

Prognosticating idiopathic pulmonary fibrosis (IPF) is challenging, in part due to a lack of sensitive biomarkers. A recent article in *Thorax* described how hyperpolarised xenon magnetic resonance spectroscopy may quantify regional gas exchange in IPF lungs. In a population of patients with IPF, we find that the xenon signal from red blood cells diminishes relative to the tissue/plasma signal over a 12-month time period, even when the diffusion factor for carbon monoxide is static over the same time period. We conclude that hyperpolarised ^129^Xe MR spectroscopy may be sensitive to short-term changes in interstitial gas diffusion in IPF.

## Introduction

Despite significant developments in our understanding of the pathogenesis of idiopathic pulmonary fibrosis (IPF) and the emergence of efficacious treatments,^1 2^ prognostication remains challenging. Pulmonary function tests (PFTs), including forced vital capacity (FVC) and diffusing capacity of the lungs for carbon monoxide (D_LCO_), form the basis of assessing progression and treatment response, but are insensitive to longitudinal change.^3^ An FVC decline of ≥10% is a validated primary endpoint for disease progression and is predictive of mortality,^4^ but longitudinal data demonstrate substantial intrapatient variability.^3^ New biomarkers may help to assess disease progression. MRI with hyperpolarised helium (^3^He) or xenon (^129^Xe) gas can reveal subtle changes in lung ventilation and microstructure.^5^ Of particular interest for pulmonary gas exchange assessment is the solubility of xenon in lung parenchyma and blood.^6 7^ ^129^Xe shows distinct signals from the red blood cells (RBCs) and interstitial tissue/plasma (TP) compartments by virtue of its environment-dependent resonant frequency, which enables evaluation of gas exchange efficiency by MR spectroscopy (MRS). Wang *et al* recently used ^129^Xe MRS to identify regions of gas transfer deficit in IPF.^8^ Herein, we describe preliminary findings evaluating the sensitivity of ^129^Xe MRS to longitudinal physiological changes in patients with IPF.

## Methods

In a study with National Health Service Research Ethics Committee approval, 18 participants with a multidisciplinary diagnosis of IPF were recruited and provided prospective informed written consent. Five patients were taking antifibrotic treatment on recruitment to the study (four on pirfenidone and one on nintedanib). During the study, a further three patients commenced pirfenidone and two patients commenced nintedanib. All underwent baseline imaging and 10 underwent repeated scans 1–3 hours later. Fourteen participants returned at 6 months (195±24 days) and 13 returned at 12 months (361±31 days). Each underwent pulmonary MRS with hyperpolarised ^129^Xe gas on a 1.5T whole-body MRI scanner. Scans were well tolerated and completed in all, except one participant at the 12-month visit, who completed neither D_LCO_, nor the MR breath-hold manoeuvre.

^129^Xe was polarised under regulatory licence and 600 mL of isotopically enriched xenon was balanced with nitrogen to a total inhaled dose of 1 L. Whole lung ^129^Xe MRS was performed during a 10–15 s breath-hold after inhaling the gas mixture from functional residual capacity. A pulse-acquire sequence was used to acquire whole-lung spectra of ^129^Xe in the TP and RBC compartments (bandwidth: 1.8 kHz; flip angle: 90°; repetition time: 1 s). Data analysis was performed using MATLAB. After discarding the first spectrum, spectra were averaged and zeroth-order phased, and integrals over the RBC and TP spectral peaks were evaluated to derive the ratio RBC:TP.

PFTs were performed on the same day as MR. Spearman’s r determined the strength of correlations. Friedman tests evaluated the significance of differences in baseline, 6 and 12-month metrics. Intraclass correlation coefficient and Bland-Altman analyses assessed reproducibility of RBC:TP. Two-tailed p values of <0.05 determined statistical significance.

## Results

**Figure 1:**
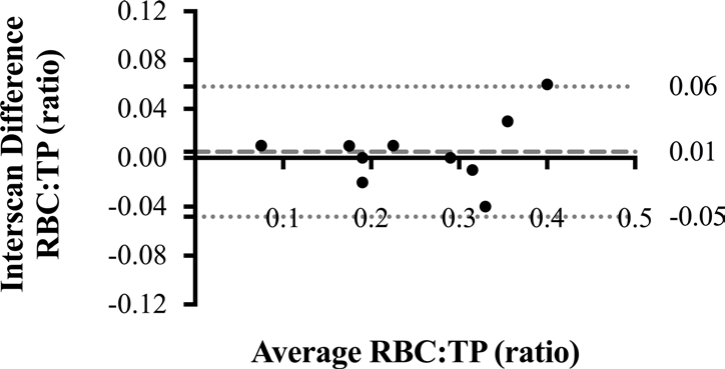
Bland-Altman plot of ^129^Xe RBC:TP at baseline. Bland-Altman plot of intraparticipant, interscan RBC:TP ratio reproducibility in 10 participants, with the y-axis representing percentage error. Dashed line represents bias, with dotted lines representing 95% CIs. RBC, red blood cell; TP, tissue/plasma.

**Figure 2:**
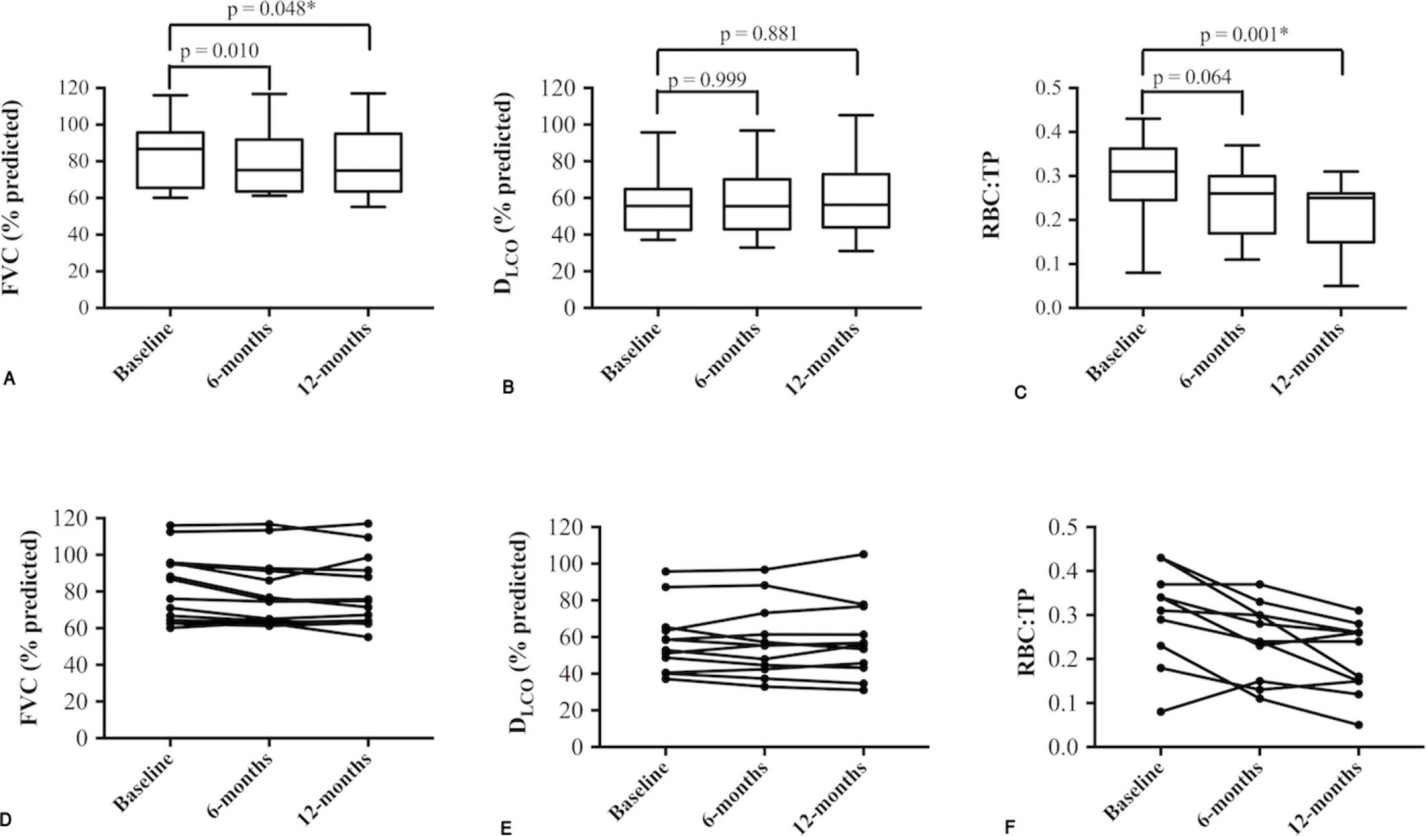
Longitudinal change in FVC, D_LCO_ and RBC:TP. (A–C): Box and whisker diagrams with associated Friedman’s test p values, representing 6-month and 12-month follow-up change in FVC, D_LCO_ and 129-xenon MR-derived RBC:TP ratio. Note that a statistically significant difference was not demonstrated in D_LCO_ (B), whereas FVC (A) and RBC:TP (C) declined significantly over 12 months. Individual participants’ metrics of FVC, D_LCO_ and RBC:TP at baseline, 6 and 12 months are plotted in (D), (E) and (F), respectively. D_LCO_, diffusing capacity of the lungs for carbon monoxide; FVC, forced vital capacity; RBC:TP, red blood cell to tissue/plasma.

Summary statistics of demographics, PFTs and RBC:TP measurements (median (IQR)) are as follows: age (years) 71.5 (67.8–74.3); FVC (%-predicted) 75.1 (64.3–95.1); D_LCO_ (%-predicted) 42.0 (34.6–56.3); baseline RBC:TP 0.26 (0.18–0.34). Same-day repeat scanning yielded highly reproducible intraparticipant RBC:TP ratios, (see Bland-Altman plot, figure 1; intraclass correlation 0.963). A statistically significant correlation (r; 95% CI) was observed between baseline RBC:TP and D_LCO_ (r=0.677; 0.293 to 0.873), but not FVC (r=0.336; −0.170 to 0.702). Correlations with D_LCO_ (%-predicted) remained significant at 6 months (r=0.831; 0.525 to 0.947) and 12 months (r=0.760; 0.312 to 0.931). Statistically significant changes (median change (95% CI); Friedman p value) were seen over 12 months in FVC (−4.2%, −7.8 to −0.2%; p=0.048) and RBC:TP (−0.08, −0.04 to −0.14; p=0.001), but not D_LCO_ (−0.5%, −5.6% to 5.8%; p=0.881), see figure 2. Although the mean observed decline in FVC was small, five patients demonstrated a relative FVC decline ≥10%, defined as clinically significant.^4^

## Discussion

Hyperpolarised ^129^Xe MRS is highly reproducible and sensitive to longitudinal changes in pulmonary gas exchange efficiency in IPF. The correlation between RBC:TP and D_LCO_ suggests the techniques reflect similar underlying gas exchange pathophysiology, although with gases that have different membrane diffusion properties. While D_LCO_ showed no longitudinal change, RBC:TP demonstrated a trend to decline over 6 months and a statistically significant decline over 12 months. Our baseline RBC:TP and D_LCO_ correlation is slightly weaker than reported in^8^ possibly due to differences in MRS parameters, reference equations for D_LCO_, or inclusion of healthy volunteer data in previous correlations.^8^

D_LCO_ is calculated from exhaled gas concentrations measured at the mouth, while MRS obtains ^129^Xe measurements directly from the alveoli, alveolar-capillary membrane and RBCs. The observed reduction in RBC:TP when D_LCO_ is stable may therefore result from differing gas properties and measurement procedure, reflecting different pathophysiology. These differences may be emphasised by the postures used when measuring D_LCO_ (sitting) and ^129^Xe RBC:TP (supine). The supine posture may cause a partial redistribution of ventilation and perfusion to regions of the lung less affected by disease. Indeed, Wang *et al* demonstrated how TP signal increases and RBC signal falls in lung tissue afflicted with IPF and how RBC signal is almost absent in areas severely afflicted.^8^ The ^129^Xe signal from the tissue and RBC compartments is weighted by a combination of specific ventilation, interstitial thickening, delayed gas diffusion and perfusion deficit. In future ^129^Xe spectroscopic studies, additional measurement the ^129^Xe alveolar gas signal as a reference, alongside contrast enhanced perfusion MRI may provide complementary physiological information to help understand the observed RBC:TP decline. Additionally, acquisition of longitudinal CT data could yield insight into the mechanism of RBC:TP change. We also note that inflation level may influence RBC:TP ratio in a non-negligible manner.^9^ Thus, future studies should consider varying the inhaled xenon gas dose with lung capacity.

Despite relatively limited participant numbers, we were able to identify a statistically significant decline in RBC:TP, indicating the power of this approach. The observed FVC decline is small, but consistent with treatment intervention studies^1 2^ and suggests a clinically significant decline in lung function and parallel gas exchange impairment. The decline observed in MRS-derived RBC:TP is underpinned by different pathophysiological mechanisms to that in FVC, and thus, RBC:TP may be additive to our current repertoire of physiological assessment tools. With further development, this technique may offer a sensitive endpoint for early-stage clinical trials.
